# A Prospective Comparative Study of Enhanced Recovery After Surgery (ERAS) Versus Conventional Protocols in Emergency Laparoscopic Appendectomies

**DOI:** 10.7759/cureus.100533

**Published:** 2025-12-31

**Authors:** Revathy Nair ES, Hemanth S Ghalige, Arunkumar Tukaram, Dhanush P, Nandhini Priya

**Affiliations:** 1 Surgery, Employees State Insurance Corporation Medical College and Post Graduate Institute of Medical Sciences and Research (ESIC MC and PGIMSR), Bengaluru, IND

**Keywords:** acute appendicitis, emergency laparoscopic appendectomy, enhanced recovery after surgery (eras), length of hospital stay, visual analog scale (vas)

## Abstract

Background

Acute appendicitis is among the most common causes of acute abdominal pain requiring surgical intervention. Laparoscopic appendectomy has become the standard of care due to its advantages in terms of reduced postoperative pain, shorter hospital stay, and early return to activity. However, optimizing perioperative recovery in emergency surgical settings remains a challenge. Enhanced Recovery After Surgery (ERAS) protocols, initially developed for elective colorectal surgeries, offer a multimodal approach to improving surgical outcomes and reducing recovery time. Recent studies have shown that ERAS can be safely and effectively adapted for emergency procedures as well.

Objectives

This study was undertaken to evaluate the effectiveness of the ERAS protocol compared to conventional perioperative care in patients undergoing emergency laparoscopic appendectomy by comparing the length of hospital stay, return to routine activities, postoperative pain (Visual Analog Scale {VAS} score), and perioperative complications between the two groups.

Methods

A prospective comparative study was conducted on 62 patients undergoing emergency laparoscopic appendectomy. The patients were divided into two groups: 31 received conventional postoperative care, and 31 followed ERAS protocols. The primary outcomes analyzed were the duration of hospital stay, time to return to normal activity, and postoperative pain (VAS).

Results

The patients in the ERAS group had significantly reduced hospital stay (1.87 ± 0.51 versus 3.16 ± 0.45 days, p < 0.001), earlier return to normal activity (3.43 ± 0.50 versus 3.97 ± 0.80 days, p = 0.003), and lower VAS scores (2.53 ± 0.51 versus 3.29 ± 0.46, p < 0.001). No complications were observed in either group.

Conclusion

The ERAS protocol is a safe and effective perioperative strategy in emergency laparoscopic appendectomy. Its implementation leads to significantly improved postoperative outcomes and can be considered for broader use in acute surgical care.

## Introduction

Acute appendicitis is the most common surgical emergency worldwide [1]. Laparoscopic appendectomy has become the standard of care for uncomplicated cases due to its association with less postoperative pain and faster recovery [2]. Perioperative care has a major role in determining surgical outcomes. Traditionally, perioperative care has been conservative, often involving prolonged fasting, delayed feeding, and the routine use of drains.

Enhanced Recovery After Surgery (ERAS) is a multidisciplinary, evidence-based approach to perioperative care, developed to improve outcomes and reduce the stress response to surgery [3]. The ERAS protocol includes early feeding, multimodal analgesia, early mobilization, and the avoidance of tubes and drains where unnecessary [4]. It has shown consistent benefits in elective procedures such as colorectal and gynecological surgeries [5,6] and, more recently, in emergency settings such as appendectomy [7,8].

Conventional perioperative care for emergency appendectomies typically includes prolonged fasting, delayed oral intake, and reliance on opioid-based analgesia. These practices differ from ERAS principles [9].

This study evaluates the effectiveness of ERAS versus conventional care in emergency laparoscopic appendectomy by comparing outcomes. including pain, hospital stay, and return to routine activity.

This paper builds upon previously reported evidence and aims to contribute data from an Indian tertiary care context [10-15].

## Materials and methods

This prospective comparative study was conducted in the Department of General Surgery at Employees State Insurance Corporation (ESIC) Medical College and Post Graduate Institute of Medical Sciences and Research (PGIMSR), Bengaluru, from May 2023 to October 2024. The patients were allocated using sealed envelope randomization into two groups: group A (conventional care) and group B (ERAS protocol), each with 31 patients.

Patients aged over 18 years, who are hemodynamically stable (systolic blood pressure {SBP}: >90 mmHg), and belonging to American Society of Anesthesiologists (ASA) grade I or II were included. Exclusion criteria comprised complicated appendicitis, the requirement of postoperative ICU care, the discovery of appendicular mass during laparoscopy, pregnancy, and chronic steroid therapy.

Complicated appendicitis was defined as gangrenous appendix, perforation, abscess formation, or peritonitis. Uncomplicated appendicitis refers to an inflamed appendix without perforation. Patients with complicated appendicitis were excluded. Patients with radiological or clinical suspicion of complicated appendicitis were excluded prior to enrolment. Intraoperative findings of complicated appendicitis were predefined as exclusion criteria; however, no enrolled patient was excluded intraoperatively. Dropouts were recorded and excluded from the final analysis.

All surgeries were performed by the surgical team who will be on duty, consisting of a team of a consultant surgeon with an experience of over 20 years, a senior resident, a junior resident, and an intern. This uniformity was followed in all the cases. Routine nasogastric tube placement was avoided in patients with uncomplicated appendicitis and was selectively used only in the conventional group when clinically indicated. Postoperative analgesia differed between groups, with the ERAS group receiving multimodal, opioid-sparing analgesia.

Preoperative evaluation included history taking, clinical examination, and relevant investigations such as ultrasound of the abdomen and contrast-enhanced computed tomography (CECT) of the abdomen/pelvis. All patients underwent emergency laparoscopic appendectomy after standard pre-anesthetic clearance.

The perioperative care for group B patients followed the Enhanced Recovery After Surgery (ERAS) protocol, which was adapted in narrative form from the ERAS® Society guidelines published as an open-access article under the Creative Commons Attribution 4.0 International License (CC BY 4.0), which is available under standard academic copyright [6]. No figures, tables, or proprietary content were reproduced. Proper citation and acknowledgment were provided. The protocol was modified to suit our institution’s emergency surgical setting: preoperatively, patients received structured counselling and written informed consent. Fasting was limited to six hours for solids and two hours for clear liquids. Intraoperatively, balanced anesthesia with short-acting agents and epidural analgesia was administered. Intra-abdominal drains were avoided. Postoperatively, nasogastric tubes were removed early, and patients were encouraged to take oral sips within hours, followed by a soft diet. Early ambulation, nausea prophylaxis, and opioid-sparing multimodal analgesia were key components. Discharge was permitted once a soft diet was tolerated and mobilization was achieved.

In the conventional group, postoperative care included opioid-based analgesia as needed, delayed oral intake after return of bowel sounds, restricted mobilization for the first 24 hours, and the removal of the urinary catheter by postoperative day 1. In contrast, the ERAS group followed preemptive multimodal analgesia, early enteral feeding within six hours, the routine avoidance of tubes and drains, and early ambulation within 4-6 hours post-surgery. Discharge criteria for both groups included pain controlled on oral analgesia, ability to tolerate diet, and independent ambulation.

The required sample size was calculated using the following formula (Cohen’s d-based two-sample t test): n = [2(Zα/2 + Zβ)² × σ²] / δ², where Zα/2 is the standard normal variate at a 5% level of significance, Zβ is the standard normal variate for power, σ is the pooled standard deviation, and δ is the minimum clinically significant difference between the group. This formula is based on the method described by Charan and Biswas, where Zα/2 = 1.96 (for 95% confidence), Zβ = 0.84 (for 80% power), σ = 1.6 (pooled standard deviation), and δ = 1.14 (expected difference in means) [16]. Substituting the values results in the following: n = [2 × (1.96 + 0.84)² × (1.6)²] / (1.14)² = [2 × 7.84 × 2.56] / 1.2996 ≈ 31 per group. Thus, a total of 62 participants (31 per group) was determined.

Postoperative pain was assessed using the Visual Analog Scale (VAS) on postoperative day 1. The VAS is a validated, generic tool consisting of a 10 cm horizontal line where 0 represents “no pain” and 10 represents “worst imaginable pain.”

Postoperative outcomes assessed include return of bowel activity, surgical site infection, readmission, postoperative pain, and ileus. The original description by Huskisson (1974) has been cited [17]. VAS is in the public domain and does not require formal permission for academic use, and no figures or diagrams were reproduced.

The study was conducted under the approval of the Institutional Ethics Committee of Employees State Insurance Corporation Medical College and Post Graduate Institute of Medical Sciences and Research and Model Hospital (approval number: 532/L/11/12). This study was not registered in a clinical trial registry, as it was an academic postgraduate thesis approved by the ethics board.

Statistical analysis

All data collected were compiled and entered into a Microsoft Excel (Microsoft Corp., Redmond, WA) worksheet. Descriptive statistics such as mean, median, mode, standard deviation (SD), and interquartile range (IQR) were calculated for continuous variables. Percentages and proportions were used for categorical variables.

Mean, median, and mode were calculated automatically using the Epi Info version 7 (CDC, Atlanta, GA) descriptive statistics module. Normality was assessed using the Shapiro-Wilk test. Quantitative variables were expressed as mean ± SD, while qualitative variables were presented as percentages. Inferential statistics were performed using the independent t-test for the comparison of continuous variables and the chi-square test for categorical variables. All analyses were conducted using Epi Info version 7. A p-value of <0.05 was considered statistically significant.

## Results

The demographic distribution between the groups was comparable in terms of age and gender. The mean age in the ERAS group was 31.2 years, while in the conventional group, it was 30.5 years, as depicted in Table 1. Gender distribution showed a similar male-to-female ratio in both groups.

**Table 1:** Demographic Characteristics of the Study Population ERAS, Enhanced Recovery After Surgery; SD, standard deviation

Parameter	Group B (Conventional, n = 31)	ERAS Group (n = 31)
Age (Years)		
Mean ± SD	30.5 ± 10.7	31.2 ± 11.9
Median	32	35
Range	10-51	10-49
Gender		
Male	17 (54.8%)	17 (54.8%)
Female	14 (45.2%)	14 (45.2%)

Postoperative outcomes were significantly better in the ERAS group. The average hospital stay in the ERAS group was 1.87 ± 0.51 days, significantly shorter than 3.16 ± 0.45 days in the conventional group (p < 0.001), as depicted in Table 2. The patients in the ERAS group resumed normal activity significantly earlier (3.43 ± 0.50 days) than those in the conventional group (3.97 ± 0.80 days) (p = 0.003), as depicted in Table 2.

**Table 2:** Perioperative Outcomes Between the Conventional Group and ERAS Group P-values calculated using an independent t-test ERAS, Enhanced Recovery After Surgery; SD, standard deviation

Outcome Measure	Conventional Protocol (Mean ± SD)	ERAS Protocol (Mean ± SD)	P-value
Duration of Hospital Stay (Days)	3.16 ± 0.45	1.87 ± 0.51	<0.001
VAS Score	3.29 ± 0.46	2.53 ± 0.51	<0.001
Return to Normal Activity (Days)	3.97 ± 0.80	3.43 ± 0.50	0.003

Furthermore, pain levels measured using the Visual Analog Scale (VAS) on postoperative day 1 were also significantly lower in the ERAS group (2.53 ± 0.51) compared to the conventional group (3.29 ± 0.46) (p < 0.001), as observed in Table 2.

No complications such as surgical site infection, readmission, or ileus were reported in either group.

## Discussion

This prospective comparative study demonstrates that the Enhanced Recovery After Surgery (ERAS) protocol significantly improves early postoperative outcomes in emergency laparoscopic appendectomy. The findings from our study, including reduced hospital stay, lower postoperative pain scores, and earlier return to routine activity in the ERAS group, are consistent with a growing body of literature supporting ERAS in emergency abdominal surgery. The patients in the ERAS group had significantly shorter hospital stays (mean: 1.87 ± 0.51 days), lower pain scores (VAS: 2.53 ± 0.51), and earlier return to routine activity (mean: 3.43 ± 0.50 days) compared to those receiving conventional care.

These findings are in agreement with previously published studies. For example, Trejo-Ávila et al. reported that the application of ERAS protocols allowed same-day discharge after laparoscopic appendectomy, confirming its role in minimizing hospital stay and expediting recovery [11]. López et al. similarly observed that patients managed under ERAS had earlier return to activity and shorter hospital stay compared to conventional care [12]. Nechay et al. demonstrated through a prospective randomized trial that ERAS implementation led to significant reductions in opioid use, improved gastrointestinal recovery, and earlier discharge following laparoscopic appendectomy [13].

These benefits of ERAS have also been observed in pediatric populations. Zhang et al. showed that ERAS implementation in children undergoing laparoscopic appendectomy significantly improved postoperative pain control and facilitated early mobilization and discharge [14]. Additionally, Ljungqvist et al. emphasized the importance of evidence-based ERAS pathways in reducing surgical stress and enhancing recovery across multiple specialties, including general surgery [15].

Although Finnerty et al. did not specifically evaluate ERAS, their findings identified key factors influencing adverse outcomes in laparoscopic appendectomy, supporting the importance of standardized care pathways in improving outcomes [4]. The present study contributes to this evolving evidence base by demonstrating the real-world impact of ERAS in the emergency surgical setting.

Our study strengthens this growing evidence, providing further support from a resource-limited, high-volume center. Despite ERAS being well-studied in elective colorectal surgery, its application in emergency surgeries remains underutilized [3].

In our study, additional recovery parameters were monitored. The patients in the ERAS group tolerated oral sips within six hours, ambulated within 12 hours, and had minimal postoperative nausea and vomiting. No readmissions or complications such as ileus or infection were recorded within 30 days post-discharge. These outcomes reflect the efficacy and safety of ERAS in enhancing functional recovery even in emergency settings.

The longer hospital stay observed in the conventional care group can be explained by the delayed initiation of oral feeding, greater reliance on opioid-based analgesia, and later ambulation. These factors are known to prolong postoperative ileus, increase pain perception, and delay functional recovery. Since both groups were comparable in demographic profile and disease severity, the differences in outcomes are best explained by variations in perioperative management rather than patient- or disease-related factors.

Multimodal, opioid-sparing analgesia reduces opioid-related adverse effects such as ileus and sedation, thereby facilitating early ambulation. Early enteral feeding stimulates gastrointestinal motility and attenuates the postoperative stress response, while the avoidance of routine tubes reduces discomfort and enhances mobility. Collectively, these physiological effects explain the improved postoperative recovery and earlier discharge observed in the ERAS group.

The results reinforce the principle that early oral nutrition, opioid-sparing analgesia, and prompt ambulation reduce surgical stress and facilitate faster recovery. Our study adds to the growing Indian data supporting ERAS protocols in nonelective abdominal surgeries.

The superior outcomes observed in the ERAS group can be scientifically explained by several physiological mechanisms. Multimodal analgesia reduces opioid requirement, thereby minimizing postoperative ileus and sedation, which supports earlier mobilization. Early enteral feeding stimulates gut motility, prevents bacterial translocation, and reduces catabolic stress. The avoidance of routine drains and nasogastric tubes reduces nociceptive stimuli, allowing improved mobility and lower pain scores. Early ambulation enhances pulmonary function, promotes bowel activity, and decreases thromboembolic risk. Collectively, these factors contribute to the shorter hospital stay and faster recovery observed in the ERAS cohort.

Limitations of the study

This study is limited by its single-center design and relatively small sample size (n = 62), which may limit generalizability, with a relatively short follow-up period, limiting the assessment of long-term postoperative outcomes. All surgeries were performed by surgeons with comparable experience in laparoscopic appendectomy, and the ERAS protocol was implemented uniformly according to institutional practice, thereby minimizing operator- and protocol-related variability. Only patients with uncomplicated appendicitis were included, excluding those with complicated disease or requiring ICU. Although the group assignment was done via the envelope method, the lack of blinding could have introduced performance bias. The study was also unblinded, introducing performance bias. Additionally, long-term follow-up beyond 30 days and quality-of-life outcomes were not assessed.

## Conclusions

The ERAS protocol significantly reduces postoperative pain and hospital stay and accelerates the return to normal activity in patients undergoing emergency laparoscopic appendectomy. By addressing the physiological stress of surgery through multimodal interventions, ERAS enhances patient recovery and overall satisfaction. Our findings reaffirm the clinical benefits of ERAS in emergency surgical settings and underscore the importance of multidisciplinary collaboration in implementing protocol-based perioperative care.

We recommend incorporating ERAS principles into routine perioperative management for emergency surgeries. Standardizing ERAS adoption in both elective and emergency cases can lead to more consistent and cost-effective patient care. Future large-scale randomized trials are needed to further validate these findings and optimize ERAS components for broader application in emergency surgical populations.

## Appendices

Declaration of permissions

Enhanced Recovery After Surgery (ERAS) protocol

The ERAS protocol used in this study was adapted in narrative form from the ERAS® Society’s 2018 recommendations published as an open-access article under the Creative Commons Attribution 4.0 International License (CC BY 4.0): Gustafsson UO, Scott MJ, Hubner M, et al.: Guidelines for perioperative care in elective colorectal surgery: Enhanced Recovery After Surgery (ERAS®) Society recommendations: 2018. World J Surg. 2019, 43:659-95 [6].

No copyrighted tables, figures, or proprietary content were reproduced. The adaptation was done strictly for academic and noncommercial research purposes. Proper citation and acknowledgment were provided wherever the protocol was mentioned.

Visual Analog Scale (VAS)

The Visual Analog Scale (VAS) used for pain assessment in this study originates from the following: Huskisson EC: Measurement of pain. Lancet. 1974, 304:1127-31 [17].

VAS is a well-established, nonproprietary tool widely accepted in clinical and academic research. It is considered to be in the public domain and does not require permission for reuse in academic studies. No copyrighted diagrams or figures were reproduced, and the original source has been appropriately cited.

This declaration is submitted to fulfill the journal’s requirement for the documentation of permission for the reuse of standardized tools in academic research.
